# Clinical Pharmacology of Vinpocetine: Properties Revisited and Introduction of a Population Pharmacokinetic Model for Its Metabolite, Apovincaminic Acid (AVA)

**DOI:** 10.3390/pharmaceutics15102502

**Published:** 2023-10-20

**Authors:** Zvonimir Petric, Paulo Paixão, Augusto Filipe, José Guimarães Morais

**Affiliations:** 1 Department of Pharmacological Sciences, Research Institute for Medicines (iMed.ULisboa), Faculty of Pharmacy, University of Lisbon, 1649-004 Lisboa, Portugal; 2 Medical Department, Tecnimede, Sociedade Técnico-Medicinal, S.A., Zona Industrial da Abrunheira, Rua da Tapada Grande, No. 2 Abrunheira, 2710-089 Sintra, Portugal

**Keywords:** vinpocetine, apovincaminic acid, pharmacology, population pharmacokinetic model, simulation

## Abstract

This paper examines the use of vinpocetine in the context of clinical pharmacology. The main and active metabolite of vinpocetine is apovincaminic acid (AVA). Due to the scarce information in the literature on AVA pharmacokinetics, we propose a population pharmacokinetic (PopPK) model for AVA based on a study in healthy volunteers with three different formulations of vinpocetine. The suggested PopPK model (and simulations) could be helpful in ensuring the more effective and safer use of the vinpocetine in the future given the increasing range of suggested indications for its use.

## 1. Introduction

Pharmacology and pharmacognosy are two related scientific fields that are traditionally studied independently, despite having shared interests in the effects of drugs on the human body. Among the various natural products that have significant pharmacological importance, alkaloids are the most frequently encountered. For instance, the plant family Apocynaceae boasts over 2500 different types of alkaloids [1]. Some of the most visually appealing plants in this family, such as Catharanthus roseus (Madagascar periwinkle), contain vinca alkaloids vincristine and vinblastine, which are well-known antineoplastic agents. Vinca minor (genus Vinca) is another plant in this family that contains vincamine, along with several other vinca alkaloids [2]. The chemical structure of alkaloids, including vinca, is modifiable, leading to the creation of new semi-synthetic and synthetic derivatives with varying pharmacological properties [3]. 

**Figure 1:**
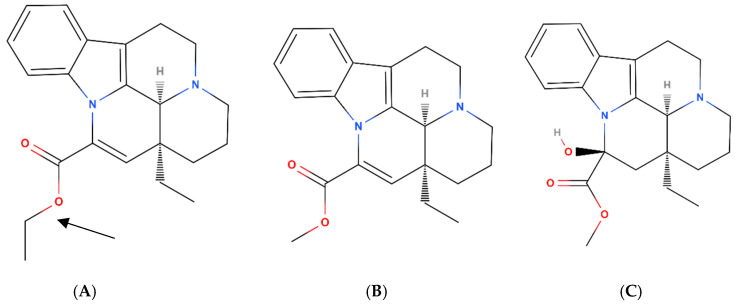
Chemical structures of vinpocetine and vinca alkaloids (from left to right: vinpocetine (**A**), apovincamine (**B**), and vincamine (**C**)). Vinpocetine is also known as ethyl apovincaminate (an ethyl group is depicted by an arrow). Figures are computed using an open-source tool [10].

Vinpocetine (Figure 1) is a semi-synthetic derivative of vincamine [4], or more precisely, it is a derivative (ethyl ester) of apovincamine which is a reaction product of heating vincamine at very high temperatures [5]. Vinpocetine was firstly marketed in Hungary as Cavinton^®^ by Gedeon Richter Ltd., Budapest, Hungary (formerly Organon), and it is one of their best-selling products in their central nervous system portfolio to this day [6]. Since the 1980s, the use of vinpocetine has spread to countries including Germany, Poland, Russia, Japan, Portugal, and others [7], where it was used as a supportive treatment in acute ischemic stroke. The main active de-esterified metabolite of vinpocetine is apovincaminic acid (AVA) (Figure 1) [8,9]. 

Nowadays, vinpocetine in the form of tablets (5 mg to 15 mg) [11,12,13,14] is claimed to support healthy brain function and improve brain blood flow, which facilitates the supply of oxygen and glucose, alleviating memory loss and reduced concentration. Some of these formulations also contain Gingko Biloba extract in combination with vinpocetine. Naturally, this immediately raises questions about the justification for such a combination and whether it has any unwanted clinical implications.

However, the use of vinpocetine as a popular dietary supplement remains controversial [15,16,17]. Despite recent renewed scientific attention [18,19,20,21,22,23], the existing literature on vinpocetine’s pharmacological properties, safety, effectiveness, and toxicity remains insufficiently comprehensive [24,25]. Our goal is to provide a pharmacological overview of vinpocetine and propose a population pharmacokinetic (PopPK) model of apovincaminic acid (AVA), an active metabolite of vinpocetine. Such a model could be useful for optimizing dosing in new indications and ensuring safer use of the drug. To our knowledge, no data on AVA in the context of pharmacometrics have been reported to date. 

## 2. Materials and Methods

### 2.1. Literature Search for Vinpocetine

To gather the most relevant pharmacological information on vinpocetine, textbooks and journal articles were consulted, and internet searches were conducted using the keyword “vinpocetine”, with sub-keywords such as “pharmacology”, “pharmacokinetics”, “metabolites”, “safety”, “danger”, “dietary supplement”, “indications”, etc.

### 2.2. Pharmacokinetic Data for Apovincaminic Acid (AVA)

Pharmacokinetic data regarding the AVA concentrations was obtained in a relative bioavailability study. This study utilized an open crossover design, in which healthy male subjects (*n* = 12) were given a single dose of vinpocetine (20 mg) from one of three formulations (#1 sustained release—Ultra Vinca^®^ (complex with β-cyclodextrin, 10 mg tablet, provided by Tecnimede, Portugal); #2 immediate release—Cavinton^®^ (5 mg tablet, Organon/Gedeon Richter, Budapest, Hungary); and #3 oral solution—5 mL/10 mg (prepared by the pharmacist at the hospital from the pure API provided by Tecnimede, Portugal)). The study included a wash-out period of 7 days after the use of each formulation. The usual approach for determining the duration of a washout period is to consider at least 5 to 10 elimination half-lives of a drug. However, in our trial, we opted for a 7-day washout period due to logistical constraints. This decision was driven by the need to coordinate the schedules of all 12 participants, which proved to be a challenging task within a shorter timeframe. Furthermore, the continuous presence of medical staff was indispensable, as they played a vital role in closely monitoring the participants for any potential adverse reactions. By implementing this 7-day washout period, we were also certain that there was no possibility of a carry-over effect. Demographic data, including race, height, and weight, were recorded for each subject, and written informed consent was obtained prior to the study. Plasma samples were collected at different time points ranging from 0.25 to 10 h after dosing to measure AVA concentrations. The analytical method was developed and validated in a prior publication [26]. 

In brief, high-performance liquid chromatography (Merck/Hitachi pump (L-6000), ultraviolet detector L-42000, and automatic injector AS-2000A, HPLC LiChrospher column Merck 60-RP-Select B 125 × 3 mm, i.d. 5 μm, equipped with LiChrospher precolumn Merck 100-RP-8, 5 μm) was used to identify and quantify AVA in plasma at 254 nm. Standard reagents, all analytical grade (Merck Life Science S.L.U.—Sigma-Aldrich, Lisbon, Portugal), included chloroform, triethylamine, sodium octanesulfate monohydrate, tetrabutylammonium hydroxide (TBAH), sodium hydroxide, 95–97% sulfuric acid, orthophosphoric acid, potassium dihydrogen phosphate, and acetonitrile. The mobile phase (acetonitrile, phosphate buffer 10 mM pH 2.8, triethylamine, and sodium octanesulfate, final pH 4.70) was used at a flow rate of 0.4 mL/min. Samples were prepared in glass tubes with Teflon sealing (16 × 125 mm) containing methanol (20 μL), primidone (20 μL) as an internal standard, and TBAH (50 μL 30 mM) and added to 1 mL of plasma. Before vortexing and stirring the samples for 10 min, 3 mL of chloroform was added to each glass tube. Afterward, all samples were centrifuged (3000 rpm at 4 °C) for 5 min. The aqueous phase was removed, and 1 mL of sulfuric acid was added to the organic phase. After a new period of vortexing, stirring, and additional centrifugation, the aqueous phase was transferred to another series of tubes in which sodium hydroxide (120 μL, 1 M), chloroform (2 mL), and TBAH (50 μL, 30 mM) were added. The samples were again stirred and centrifuged, and the aqueous phase was removed so that the organic phase could be evaporated (35 °C) in a water bath under a stream of nitrogen. Finally, the dried residue, after redissolving in the mobile phase (100 μL), was injected. Regarding the analytical validation, i.e., linearity of the analytical method, solutions of different concentrations of AVA in duplicates (Merck, Sigma-Aldrich, Lisbon, Portugal) were prepared by adding known amounts of standard to blank plasma. After linear regression of the obtained data, a linearity of response was verified for the ratio of peak heights of AVA in relation to primidone peaks and for the plasma concentrations of AVA (range 5–300 ng/mL, R = 0.999). The accuracy evaluated by the calibration curves was expressed as the R.S.D. of 7%. The limits of quantification were 5 ng/mL (based on the analysis of 5 replicates of different concentrations of AVA). 

The study was conducted in accordance with the Declaration of Helsinki guidelines, ICH GCP (Good Clinical Practice), GLP (Good Laboratory Practice), and EU/Portuguese local regulations. Ethical approval was obtained from the Ethic committee of the Hospital Pulido Valente (Lisbon, Portugal).

### 2.3. Population Pharmacokinetic Model for Apovincaminic Acid (AVA)

Population PK analysis (PopPK) of AVA was performed using a non-linear mixed effect (NLME) modeling approach within the Lixoft Suite software, i.e., Monolix (version 2021R2, Lixoft SAS, a Simulations Plus company), via the stochastic approximation expectation maximization (SAEM) algorithm [27]. Simulations were made based on the optimized model using Simulx (version 2021R2, Lixoft SAS, a Simulations Plus company).

The PopPK model development procedure was evaluated using three criteria: (1) reduced estimated log-likelihood (-2 log-likelihood objective function value (OFV) for hierarchical models) and information criteria (corrected Bayesian Information Criteria (BICc)), (2) goodness-of-fit (GOF) plots, and (3) visual prediction checks (VPCs). All three formulations of vinpocetine were jointly modeled to characterize the AVA exposure.

## 3. Results and Discussion

### 3.1. Literature Search for Vinpocetine

A PubMed search using the keyword “vinpocetine” yielded 843 results, which seems limited considering the extensive timeframe of publications spanning from 1977 to 2023. Noteworthy years with significant research output include 1990 (28 publications), 2007 (31 publications), and 2020 (35 publications), indicating growing interest in vinpocetine over the decades. The majority of these publications explore the vasodilating, anti-inflammatory, and neuromodulating properties of vinpocetine in relation to various diseases. Furthermore, most studies employ in vitro, ex vivo, and animal models. Hungarian researchers have made significant contributions to the pharmacokinetic characterization of vinpocetine. In this paper, we did not include details regarding the molecular pharmacology of vinpocetine.

### 3.2. Pharmacokinetic Data for Apovincaminic Acid (AVA)

**Figure 2:**
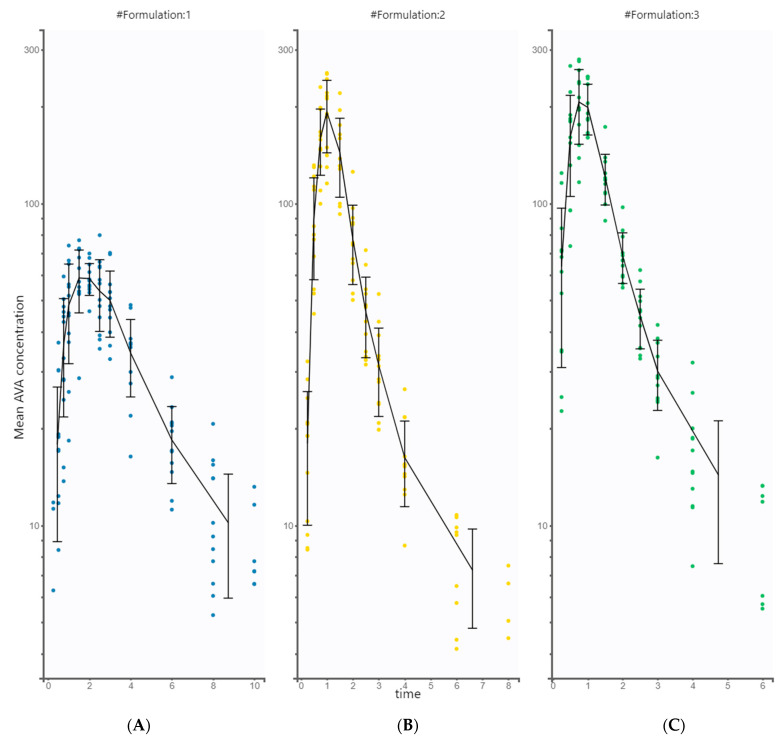
Measured mean plasma concentrations of AVA (formulations #1, #2, and #3, plots (**A**), (**B**), and (**C**), respectively) with error bars (standard deviation) on a log-linear scale. (Concentration of AVA in μg/L, time in h).

The relative bioavailability study was completed by all volunteers following the protocol, and no significant negative effects were observed. Plasma concentrations were determined for the three different formulations (#1, #2, and #3, plots A, B, and C, respectively), resulting in the mean plasma (with SD) profiles (Figure 2).

### 3.3. Population Pharmacokinetic Model for Apovincaminic Acid (AVA)

The PK model was made based on the full data available for each individual subject and formulation. No data were removed from the model, and a few concentration values of AVA below the limit of quantification were not censored.

Initially, a one-compartment model was tried with first-order absorption, linear elimination, with combined and proportional error. However, a two-compartment model with proportional residual error was found to better describe the data, although still with some misspecifications. To address this, zero-order input (Tk0) was introduced, which improved the minimization criteria, GOF and VPC plots. In addition, a model with a transit compartment was also attempted but showed no improvements. Finally, a lag time before absorption (Tlag) was added to the structural model, leading to overall improvement. 

The model was refined by introducing correlations between random effects of clearance (CL/F) and the volume of distribution of the central compartment (V1/F), while the covariate relationships were evaluated using a stepwise covariate modeling approach and selected based on statistical tests (Pearson’s test and Wald test), software proposals (based on a linear regression), minimization criteria, and physiological plausibility. We inspected age, height, weight (expressed as BMI), and formulation (as a categorical covariate) as potential covariates. However, none of the continuous covariates showed a significant impact on parameter variability, while formulation had the most significant effect (denoted as coefficient β; beta) on Tk0 and V1/F. 

**Figure 3:**
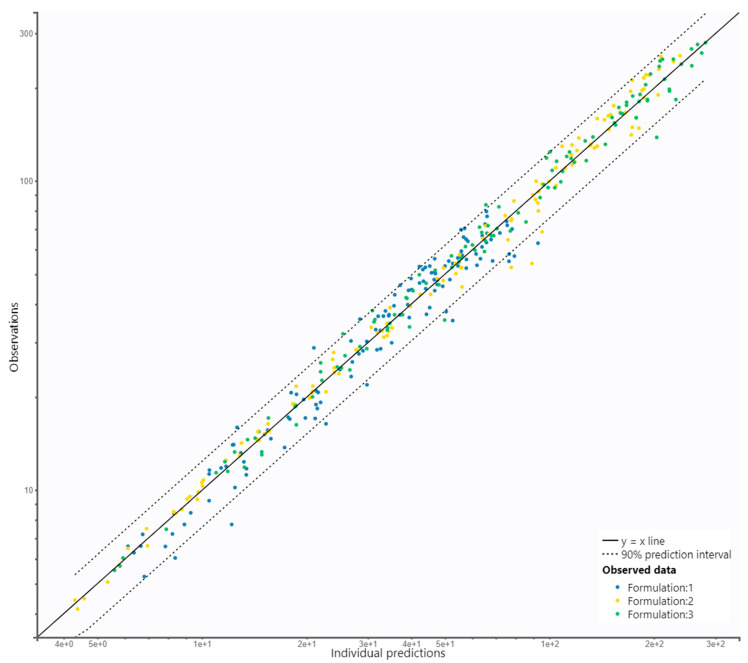
GOF plot indicating that the individual predictions fitted well along the identity line relative to observed concentrations (joined data for all three formulations of vinpocetine). Dashed line is 90% prediction interval.

**Figure 4:**
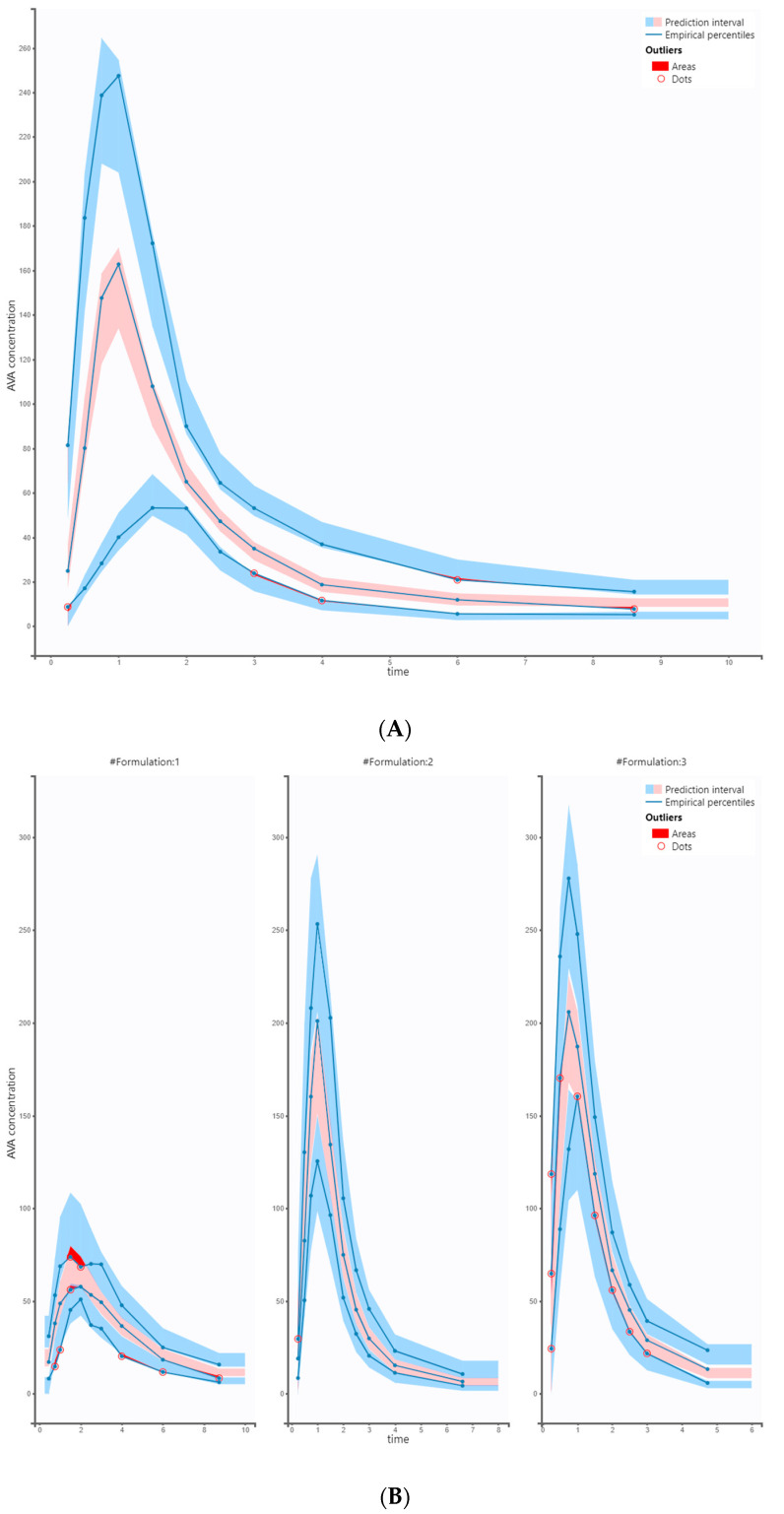
Plot (**A**): Joined VPC of AVA. The figure presents the VPC with the prediction intervals for the 10th, 50th, and 90th percentiles. The model describes the observed data well, and model predictions are within the prediction interval. Plot (**B**): Stratified VPC plots by formulations (#1, #2, #3). Minor outliers were not deemed significant, as the overall model captures the data well. (Concentration of AVA in μg/L, time in h).

Hence, the final proposed PopPK model characterizing AVA exposure in healthy subjects, based on three formulations of vinpocetine, is a two-compartment model with zero-order input and Tlag, linear elimination, and proportional residual error. Additionally, the model was refined to include correlations between random effects of V1/F and CL/F. None of the continuous covariates had a relevant impact on explaining the inter-individual variability. The final results and diagnostic plots confirm that the model accurately captures the observed data (Figure 3 and Figure 4A,B). The final model parameters are summarized in Table 1.

### 3.4. Vinpocetine—What Do We Know So Far?

The anatomical therapeutic chemical (ATC) code (assigned by the World Health organization) for vinpocetine is N06BX18, which means it is classified as a drug with an influence on the nervous system (N). It falls under the group of psychoanaleptics (06) and the subgroup of psychostimulants; agents used for attention deficit hyperactivity disorder (ADHD) and nootropics (B) [28,29]. Unlike vinpocetine, its precursor vincamine has a different ATC code, which is C04AX. This indicates that vincamine is classified as a substance used for the treatment of cardiovascular conditions (C) and belongs to the group of peripheral vasodilators (04) [30].

Vinpocetine’s most well-documented clinical use (indirect) has been in acute ischemic stroke, where it has been characterized as a neuroprotective agent. Over ten trials, including more than five randomized controlled trials, have been conducted in the past, and researchers have provided systematic reviews on vinpocetine’s effectiveness [31,32,33,34,35]. However, the final verdict on its effectiveness still remains unclear.

According to Panda et al. [24], although vinpocetine shows promising results, there is no clear evidence of reduced mortality (as a direct clinical outcome) associated with its use in such patients. Therefore, new trials with sufficient power must be performed to further investigate its potential. It is worth mentioning that in some of these trials [32], vinpocetine was administered intravenously in doses of 30–40 mg per day for several weeks, and overall, the administration of vinpocetine in stroke patients did not raise any safety concerns then.

On the other hand, although causality was not established in some cases, the literature still reports adverse effects related to the general use of vinpocetine, including both the oral and intravenous routes. These adverse effects include flushing, transient changes in blood pressure (both hypotension and hypertension), tachycardia, decreased pulse rate, dizziness, feeling of warmth during intravenous administration, cold hands and feet, vomiting, diarrhea, heartburn, urticaria, and dry mouth, as well as changes in hemoglobin and hematocrit levels, or increased γ-glutamate pyruvate transaminase, etc. Furthermore, serious adverse effects reported in some cases included atrial fibrillation, multifocal extrasystoles, epileptiform convulsions, and agranulocytosis (which resolved after proper treatment was given and vinpocetine was discontinued) [36]. Due to the structural resemblance of vinpocetine to vincamine, which is a vasodilator, it is expected that some of the previously mentioned adverse effects could occur.

The neuroprotective effects of vinpocetine are attributed to its antioxidant properties, which have been confirmed in vitro and in vivo [37,38,39,40], as well as its modulation of sodium [41] and calcium levels in neuronal cells [42]. In addition, an anticonvulsant effect has also been proposed due to sodium channel blockage and the inhibition of glutamate release [43,44]. A recent study [8] examined the effect of vinpocetine in patients with epilepsy, as well as in healthy subjects. The conclusion was that no significant benefits were observed in cognitive skills (in both groups). However, vinpocetine exposure was lower than it was expected when compared to effective dose in animals. On the contrary, based on their trial, Zhang et al. [31] concluded that patients with stroke who received vinpocetine treatment had improved cognitive skills. Moreover, they had improved cerebral blood flow, improved neurological functions, and better quality of life compared to the control group without vinpocetine treatment. Currently, there is an ongoing trial in patients with epilepsy (sponsored by the University of Stanford and with estimated completion by the end of 2024) [45]. This study investigates the potential cognitive-enhancing effects of vinpocetine in both healthy individuals and patients with epilepsy. The study aims to determine the safety, effectiveness, and blood levels of acute and chronic oral doses of vinpocetine. Specific objectives include measuring cognitive functions and memory in healthy volunteers, assessing the preliminary effectiveness of vinpocetine in enhancing cognitive functions in epilepsy patients, determining blood levels for its anticonvulsant effects, and investigating if vinpocetine can reduce seizure frequency or duration in patients with epilepsy.

In addition to its suggested neuroprotective, antioxidant, and anticonvulsant properties, vinpocetine is also suggested to have anti-inflammatory effects both in vitro and in vivo [46,47,48]. Namely, evidence shows that vinpocetine can inhibit the activation of proinflammatory mediators mediated via the tumor necrosis factor alpha (TNF-α) signaling pathway in various cell types, including macrophages, endothelial cells, and vascular smooth cells. As TNF-α is the main pharmacological target in many diseases, such as inflammatory bowel disease (Crohn’s disease and ulcerative colitis) [49], these findings should be further researched and tested in disease models that have inflammation in etiopathogenesis. For instance, in a mouse model of acetic acid-induced colitis, the administration of 30 mg/kg of vinpocetine demonstrated multifaceted properties, including anti-inflammatory, antioxidant, and analgesic effects. Vinpocetine exhibited a notable reduction in colon edema, myeloperoxidase activity, macroscopic and microscopic damage scores, as well as acetic acid-induced visceral hyperalgesia, oxidative stress, cytokine production, and downstream NF-κB activation [50]. Some attempts have also been made in the research of atherosclerosis [51], where vinpocetine was suggested to modulate many steps in the progression of the disease, due to its potential to inhibit the inflammatory signaling cascade and proinflammatory cytokines and factors. Similarly, vinpocetine was found to have beneficial effects on diabetes-associated renal damage in a rat model [52]. While clinical studies on the effects of vinpocetine on these specific cases in humans are limited, some data are also suggesting beneficial potential in treating diabetic neuropathy in patients with type 2 diabetes mellitus [53].

Given the many attributes of vinpocetine as a drug, researchers have questioned whether it has the potential to be used in treating diseases such as Parkinson’s disease [54,55], as well as dementia in older people and Alzheimer’s disease [56,57,58,59]. Parkinson’s disease is a highly intricate condition that still has an unknown etiology. Despite significant advancements in research and therapy, there is no definitive cure for the disease, and so far, all available therapeutic approaches have limitations and become ineffective over time. Although the number of clinical trials in these conditions with vinpocetine is limited, the results suggest that it can modulate molecular signaling pathways related to Parkinson’s disease, reduce levels of inflammatory cytokines and signaling molecules, and improve cognitive impairment in study participants. In contrast, clinical data on the effects of vinpocetine in Alzheimer’s disease were more extensively studied in the past, particularly before the 1990s, and some studies at that time suggested positive associations with the use of vinpocetine. However, a review by the Cochrane Database of Systematic Reviews found many inconsistencies in the evidence, and therefore, the overall evidence for the effectiveness of vinpocetine in Alzheimer’s disease remains inconclusive (up to 2003). Recently, a small clinical study in 2014 [60] reported that vinpocetine was able to improve memory and concentration in people with dementia. However, the improvement was characterized with minimal effect (when compared to the results of patients with epilepsy). Hence, considering the suggested benefits of vinpocetine, it is evident that further research in this area is necessary to fully understand its effects.

Another area where vinpocetine was also researched was ophthalmologic disorders of vision and visual field [61]. Specifically, clinical studies have indicated that vinpocetine may provide the most significant benefits for individuals with macular degeneration, while more modest improvements have been observed in patients with glaucoma and ischemia of the optic nerve. In the patient information leaflet (PIL) of Cavinton^®^ [62], indeed, one of the indications (besides the management of psychological or neurological symptoms caused by a disorder of cerebral circulation) is also the management of eye diseases that have vascular disorders as their underlying cause. For optimal results, it is recommended to take vinpocetine orally in a daily dosage of 15–30 mg, and after a meal. In addition, a recent study conducted on an ex vivo model of retinal ischemia also demonstrated a potential protective effect of vinpocetine [63].

Given the various pharmacological implications of vinpocetine outlined above, it is evident that the unregulated sale of this compound as a dietary supplement should be approached with caution. Manda et al. [64] explored the potential of vinpocetine for drug–drug interactions (DDIs), i.e., cytochromes P450 (CYPs) and P-glycoprotein (P-gp) inhibition. Vinpocetine was shown to be a strong inhibitor of P-gp (EC50 8 μM), and a moderate inhibitor of recombinant CYP3A4 (IC50 2.8 μM) and CYP2D6 (IC50 6.5 μM). Although the clinical relevance of these findings has not yet been fully explored, P-gp inhibition due to drug–drug and food–drug interactions [65] can significantly alter the pharmacokinetics (PK) and pharmacodynamics (PD) of other drugs, especially those with a narrow therapeutic index. Therefore, further investigation into the potential implications of these findings is warranted.

In light of these potential implications and as a safety precaution, there have been several attempts to re-examine vinpocetine as a dietary supplement in the USA (since 2016). Finally, in 2019, the FDA issued a safety warning for women of childbearing age regarding possible risks associated with vinpocetine, due to indications of adverse effects on reproductive health (i.e., reproductive and developmental toxicity in animals), including miscarriage and harm to fetal development [66], but the FDA has not yet issued a final decision on the matter.

In a paper from 2016 [17], researchers analyzed the amount of vinpocetine in various dietary supplements that are sold commercially. The findings showed that there was a broad range of amounts which varied considerably, with the quantity of vinpocetine per serving ranging from 0.6 to 5.1 mg in different products. Moreover, most of the supplements did not provide any information regarding the amount of vinpocetine present. The same paper stated that vinpocetine has been banned as a cognitive-enhancing supplement in Australia, New Zealand, and Canada. However, supplements with labeled amounts of up to 15 mg per tablet can be seen and purchased online [67].

It is important to recognize that the unregulated sale of nootropic drugs, including dietary supplements containing vinpocetine, is a concerning issue. These drugs are commonly known as “smart drugs” and are marketed for their ability to enhance cognitive processes such as thinking, learning, and memory. However, their use can also potentially lead to addiction. Furthermore, the long-term effects of using nootropic drugs are not yet fully understood, emphasizing the importance of close monitoring by a healthcare professional [68].

Furthermore, it should be noted that there is currently insufficient reliable information to determine the safety of using vinpocetine during breastfeeding as well. Therefore, caution is particularly important for nursing mothers (who might be unaware of dangers) until further research is conducted. In addition, the updated (2020) patient information leaflet (PIL) for Atreiza^®^ (vinpocetine) [69] emphasizes the need for necessary precautions when administering vinpocetine to individuals taking medication for hypertension, cardiac arrhythmias, or those with a medical history of QT interval prolongation. Caution is advised for individuals taking anticoagulant medications due to the potential risk of adverse effects when combined with vinpocetine. However, it appears that all of these precautions are often overlooked or disregarded when vinpocetine is marketed and sold as a dietary supplement, which clearly demonstrates the urgent need for global regulation in the field of nutrivigilance as well.

### 3.5. Population Pharmacokinetic Model of Apovincaminic Acid (AVA)

The PK of vinpocetine, including its main active metabolite, AVA, was studied a few decades ago, both in animals (dog and rat) [70,71] and humans [9,72,73]. Regarding the PK of vinpocetine in humans, the absorption of vinpocetine after an oral dose is considered fast, with the maximum concentration (Cmax) being reached within 1.5–2 h, and the elimination half-life being mostly around 1–2.5 h (depending on the study report). Oral bioavailability ranges significantly from 7% to 60%, and it is greatly influenced by food intake. Vinpocetine is metabolized in the liver, and it is considered to be very rapidly transformed [73,74]. It is noteworthy that in a particular study [72], the elimination half-life of the substance (after an intravenous (i.v.) administration) was discovered to be 4.7 ± 2.13 h, which is almost twice the typical reported duration. The pharmacokinetics of vinpocetine was estimated using the compartmental analysis (CA), which suggested that the three-compartment open model (which suggested two distribution phases) was the best fit for the data [72]. Conversely, other studies found two-compartment models to best describe the PK of vinpocetine [75].

As the biotransformation of vinpocetine to AVA occurs rapidly, measuring the maximum concentrations of vinpocetine after oral administration has been quite challenging in the past, given its short half-life and a large variability in Cmax across individuals. Consequently, there is a limited number of bioavailability/bioequivalence studies of vinpocetine reported in the literature. Therefore, AVA has been used as a surrogate of the therapeutic bioequivalence [73].

Previous studies have demonstrated that 20–40% of the administered dose of vinpocetine is converted to AVA. AVA was reported to have an elimination half-life of 3.9 ± 1.6 h and a clearance estimated at 0.09 L/h per kg of body weight assuming a 30% conversion factor from vinpocetine administered i.v. [72]. In addition, after the oral administration of vinpocetine, AVA showed a high volume of distribution (Vd/F = 114 L) and a fast elimination (t1/2 = 1 h) [73]. The PK of AVA was characterized using a two-compartment open model in animals only in certain studies [71,76], whereas in most cases, its PK parameters were estimated using non-compartmental analysis (NCA). AVA shares neuroprotective and nootropic effects with vinpocetine (confirmed in vivo) and it can also cross the blood–brain barrier (BBB) [77].

To the best of our knowledge, our paper presents the first PopPK model characterizing AVA pharmacokinetics. The average CL/F, with a value of 56.15 L/h, is in the order of previously described values for AVA. This value shows quite reasonable between-subject-variability (BSV), in the order of 21%. The volume of distribution at steady state (V1/F + V2/F) is considered to be large, e.g., for formulation#2, around 135 L. However, based on the described vinpocetine bioavailability and conversion to AVA, the expected final value should be around 21 L, indicating distribution between the extracellular and total body water, with a BSV in the order of 39%.

The disappearance of the drug during the plasma collection scheme is governed by both the distribution half-life predicted by the model, with a value of around 0.6 h, and by the terminal elimination half-life that is predicted to be around 4.7 h (for formulation#1). These values go in line with the known behavior of AVA. Regarding the covariates, only the type of formulation was considered significant. This was somewhat expected as the used population was relatively homogenous in terms of age, sex, and body weight. Although representing only a subset of the general population, this is a relevant subset of subjects that are using vinpocetine as dietary supplements. As such, this model can be used for simulating drug plasma concentrations in various scenarios, from the usual clinical use to other potential administration schemes.

In this regard, there are several suggested dosing regimens for vinpocetine, both as a drug and as a dietary supplement. Typically, the manufacturer-recommended therapeutic regimen involves an initial loading dose of one to two 5 mg vinpocetine tablets, followed by a maintaining dose of 5 mg three times a day, which equates to 15 mg per day [78]. However, when considering dietary supplements, the proposed regimes are much more liberal, and even regimens such as 15 mg three times a day (i.e., a total of 45 mg per day) exist [67].

**Figure 5:**
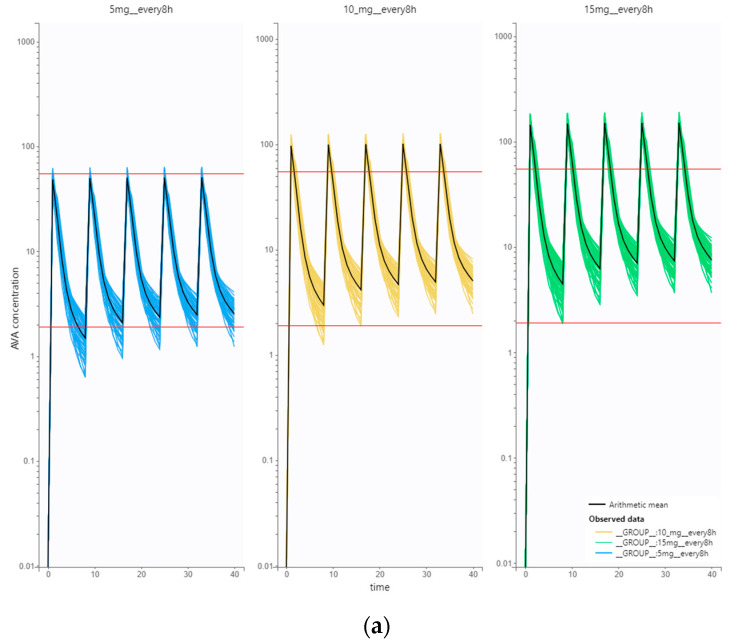

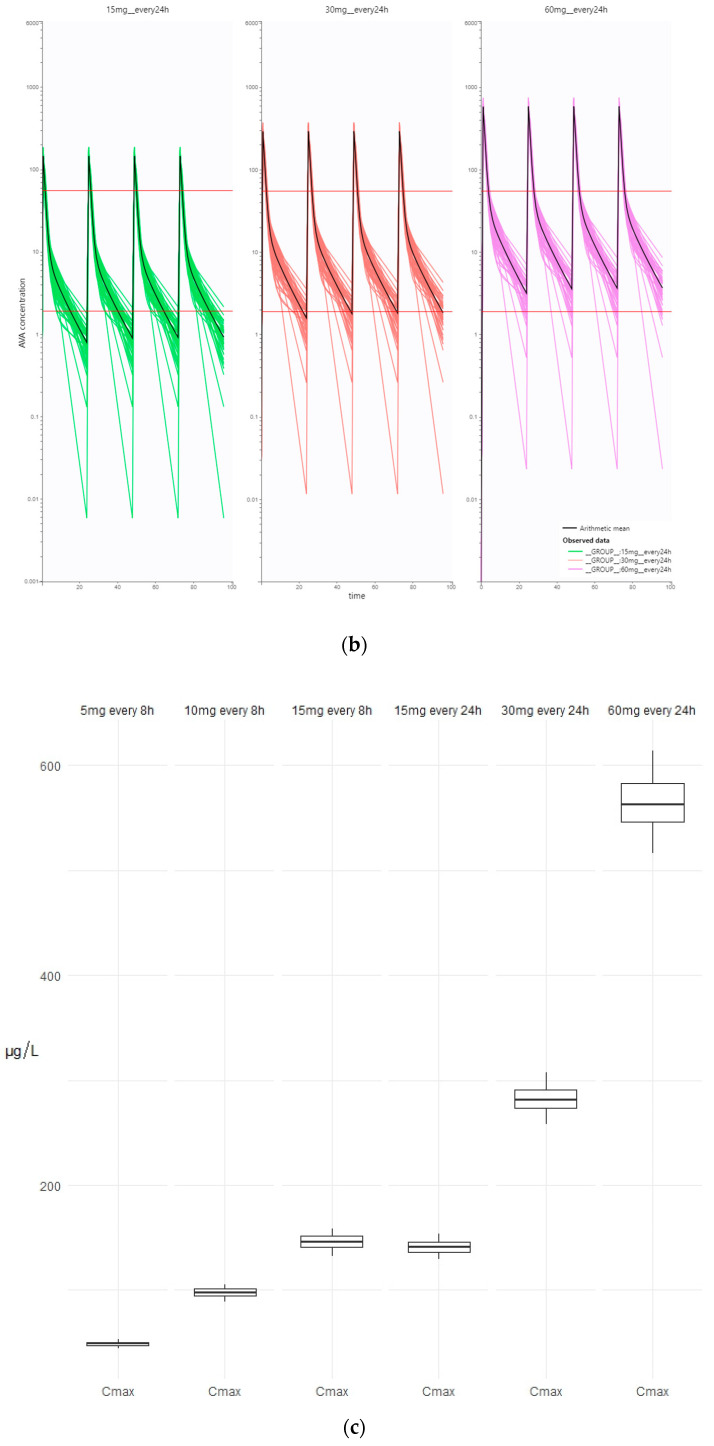

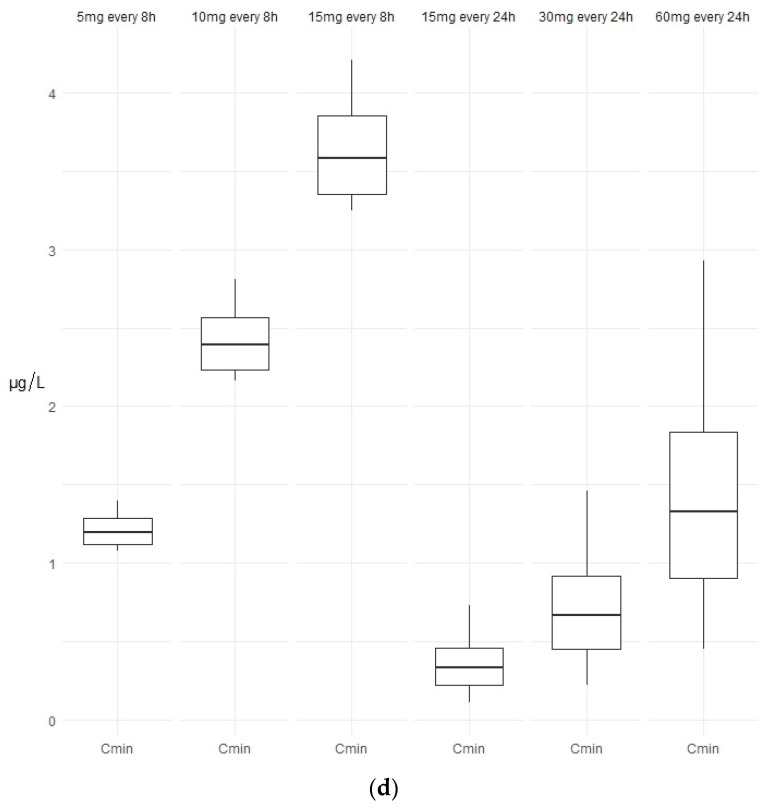
(**a**). Simulated AVA concentration vs. time plots from 50 virtual individuals (i.e., from one virtual trial replicate) who took vinpocetine tablets (5 mg/10 mg/15 mg) three times a day (every 8 h). The red line marks the concentration range between the lower 5% of Cmin values and the higher 95% of the Cmax values, which are considered to be safe and efficacious based on the clinical therapeutic regimen of 5 mg three times a day. (Concentration of AVA in μg/L, time in h). (**b**). Simulated AVA concentration vs. time plots from 50 virtual individuals (i.e., from one virtual trial replicate) who took vinpocetine tablets (15 mg/30 mg/60 mg) once a day (every 24 h). The red line marks the concentration range between the lower 5% of Cmin values and the higher 95% of the Cmax values, which are considered to be safe and efficacious based on the clinical therapeutic regimen of 5 mg three times a day. (Concentration of AVA in μg/L, time in h). (**c**). Summary statistics of the AVA Cmax values based on the simulated dosage regimens of vinpocetine from ten trial replicates. The concentration of AVA on the *y*-axis is expressed in micrograms per liter (μg/L). (**d**). Summary statistics of the AVA Cmin values based on the simulated dosage regimens of vinpocetine from ten trial replicates. The concentration of AVA on the *y*-axis is expressed in micrograms per liter (μg/L).

Also, some individuals who self-identify as “biohackers/neurohackers” report taking up to 60 mg of vinpocetine in a single dose, and certain websites dedicated to nootropics suggest that taking up to 60 mg of vinpocetine per day is safe [79]. As there is evidence that AVA is the primary mediator responsible for enhancing neuronal excitability [80], we conducted simulations using Simulx (Figure 5a,b) to gain insight into the plasma concentration differences between these commonly suggested regimens. The simulated dosage regimens were either multiple doses every eight hours or once per day as a single dose. We conducted simulation with ten trial replicates with 50 virtual individuals in each, also accounting for the uncertainty around the estimated PopPK parameters for AVA. Our post-processing simulation goal was to examine the differences in Cmax and minimum concentration (Cmin) values among the simulated dosing regimens from all trial replicates (Figure 5c,d). Since there is no established exposure–response relationship for AVA, we considered that the lower 5% concentrations obtained with the maintenance therapeutic regimen of vinpocetine as a drug (5 mg three times a day) are required for minimal effectiveness and that the higher 95% concentration in the same regimen limits the safety margin. Simulation summary statistics (Figure 5c,d) were postprocessed graphically using R software (version: 4.3.1 (16 June 2023), R Foundation for Statistical Computing, Vienna, Austria).

The simulations showed that taking vinpocetine multiple times a day resulted in lower mean Cmax values of AVA, as expected, compared to once-daily dosing. For instance, a regimen of 5 mg every 8 h showed a mean AVA Cmax of 48.7 μg/L, whereas a regimen of 15 mg every 24 h showed a much higher mean Cmax of 140.7 μg/L. Additionally, there were notable differences in Cmax values among virtual individuals, depending on the specific regimen. In the latter example, Cmax ranged from 44.21 μg/L (minimum) to 52.6 μg/L (maximum) for the 5 mg every 8 h regimen, and from 129 μg/L (minimum) to 153.5 μg/L (maximum) for the 15 mg every 24 h, respectively. The highest degree of differences in Cmax was observed with the highest simulated daily dose (Figure 5c). It is also important to note that the 60 mg once a day regimen resulted in Cmax values that were 10 times greater than the usual values obtained with the therapeutic regimen of 5 mg every 8-h.

Regarding the mean AVA Cmin values, it is evident that the once-daily regimen of AVA consistently resulted in lower Cmin values. For example, the 5 mg every 8 h regimen had a mean Cmin of 1.25 μg/L, while the 15 mg every 24 h regimen had a mean Cmin of 0.52 μg/L, which is approximately twice as low (Figure 5d). All other simulated regimes resulted in Cmin at or above the 5 mg every 8 h regimen; however, they also carried an increased risk of significantly higher Cmax values.

While it is commonly believed that vinpocetine is safe, the latest experiments on animals have shown otherwise in regard to reproductive toxicity. Also, there may still be important pieces missing from the puzzle that we are not yet aware of, such as DDIs or off-target effects, which could have significant implications for the safe use of vinpocetine. As such, it is crucial to establish the efficacy and potency of vinpocetine and AVA and determine the appropriate way to quantify both primary and secondary pharmacology. Furthermore, it is important to note that the bioavailability of vinpocetine can be influenced by food intake, particularly a high-fat meal. Therefore, the differences in Cmax values observed between different dosing regimens may have unknown clinical implications. This is especially concerning for consumers of dietary supplements, who may not be aware of how different dosing regimens containing the same daily dose can lead to different PK and ultimately affect the effectiveness and safety of the supplement.

In conclusion, this simulation demonstrates the potential concerns of effectiveness as well as safety associated with a flexible dosing window of vinpocetine without the supporting clinical evidence, particularly when dosing recommendations lack the context of a PKPD relationship.

Our study had limitations which include a small sample size and a narrow range of covariates in the study cohort (see Supplementary Material). One must also note the variable AVA plasma concentrations among individuals. To gain a more comprehensive understanding of vinpocetine’s (and AVA’s) exposure and effects, future studies should include larger sample sizes and more diverse patient populations. Additionally, testing different dosing regimens and using measurable endpoints may provide new insights into the dose–exposure–response relationship of vinpocetine in patients. It is important to note that while metabolites may not follow the same PK behavior as their parent drugs, they can still exhibit the same PD as the parent drug. Finally, detailed insights into AVA’s molecular and clinical aspects can provide a more accurate understanding of its pharmacological profile, including its potency, efficacy, and effectiveness.

## 4. Conclusions

Vinpocetine is a drug with a very diverse pharmacological profile, with possibilities of interacting with multiple pharmacological targets. Thus far, vinpocetine has mostly been suggested for use in neurological and cerebrovascular disorders, as well as in some eye-related disorders. It has also been shown to have vasodilating and antioxidant effects. However, based on in vitro and in vivo animal models, vinpocetine has shown potential for use in various inflammation-related and cardiovascular/vascular disorders. Although there has been some research on vinpocetine and its potential effectiveness in treating epilepsy, dementia, Parkinson’s, and Alzheimer’s disease, it is still too early to draw scientifically strong and valid conclusions. More unbiased clinical trials with good power are needed in the future to validate the effectiveness of vinpocetine in treating these diseases and to determine any newly suggested benefits, no matter how promising the scientific evidence regarding vinpocetine may sound.

The sale of vinpocetine as a nootropic dietary supplement both online and in stores should be strictly regulated due to concerns about its safety for consumers. Animal models have demonstrated reproductive and developmental toxicity associated with the use of vinpocetine, which raises questions about the potential risks to humans. Furthermore, the unknown potential for clinically relevant DDIs should also not be overlooked.

Apovincaminic acid (AVA) is a major and active metabolite of vinpocetine, often used as a surrogate for exposure to vinpocetine. A population pharmacokinetic model of AVA on twelve healthy male individuals and three formulations of vinpocetine showed that AVA exposure can be modeled as a two-compartment model with zero-order input (Tk0) with Tlag and linear elimination.

Making recommendations for a flexible dosing window of vinpocetine without supporting clinical evidence, particularly when the dosing recommendations lack the context of the PKPD relationship, requires careful thought and consideration to ensure vinpocetine’s effectiveness and safety.

## Figures and Tables

**Table 1:** Estimated parameters of the PopPK model of AVA., i.e., estimated population parameters, inter-individual variability, and residual error parameter.

Stochastic Approximation			
		S.E.	R.S.E. (%)
Fixed Effects			
Tlag_pop h	0.17	0.013	7.66
Tk0_pop h	1.35	0.12	8.87
β_Tk0_Formulation#2	−0.4	0.12	29.0
β_Tk0_Formulation#3	−0.68	0.13	18.5
Cl/F_pop L/h	56.15	2.15	3.83
V1/F_pop L	208.34	11.07	5.31
β_V1/F_Formulation#2	−1.26	0.068	5.44
β_V1/F_Formulation#3	−1.24	0.071	5.71
Q/F_pop L	14.63	1.7	11.6
V2/F_pop L	76.15	11.38	14.9
**Standard Deviation of the Random Effects (omega)**			
ω_Tlag	0.38	0.082	21.4
ω_Tk0	0.24	0.036	14.8
ω_Cl/F	0.21	0.027	13.0
ω_V1/F	0.15	0.034	22.4
ω_Q/F	0.47	0.1	21.1
ω_V2/F	0.24	0.093	39.1
**Correlations**			
corr_V1/F_Cl/F	0.72	0.14	19.0
**Error Model Parameters**			
b	0.14	0.0076	5.28

Abbreviations: S.E.—standard error, R.S.E—relative standard error, Tlag_pop—estimated lag time before absorption, Tk0_pop—estimated zero-order input, β—estimated coefficient accounting for categorical covariate (i.e., formulation when a population parameter shows dependency on a covariate), CL/F—estimated apparent clearance when the ratio of clearance to bioavailability (F) is assumed constant, V1/F, V2/F—estimated apparent volume of distribution accounting for the first and second compartment, respectively, Q/F—estimated intercompartmental clearance between compartments, corr—correlation between population parameters, b—parameter of the error model for the observations.

## Data Availability

The general data presented in this study are available upon request from the corresponding author. Please note that some information may not be fully available due to privacy protection.

## Supplementary Materials

The following supporting information can be downloaded at: https://www.mdpi.com/article/10.3390/pharmaceutics15102502/s1. Table S1: Demographic data (number of subjects = 12); Table S2: Change in estimated log-likelihood and information criteria (from top to bottom: final refined model, best structural model, and initial starting model).
