# Supplementary material for: Clinical Pharmacology of Vinpocetine: Properties Revisited and Introduction of a Population Pharmacokinetic Model for Its Metabolite, Apovincaminic Acid (AVA)
Source: Pharmaceutics. 2023 Oct 20;15(10):2502. doi: 10.3390/pharmaceutics15102502 (PMC10610279; doi:10.3390/pharmaceutics15102502)
Supplement: Supplementary file 1 [file pharmaceutics-15-02502-s001.zip › pharmaceutics-2659907-supplementary.pdf]

Supplementary Materials

Table S1. Demographic data (Number of subjects = 12).

	MIN	*MEDIAN*	*MAX*
Age	20	23	35
BMI (kg/m²)	20.9	24.9	32.56
Height (m)	1.64	1.74	1.83
Weight (kg)	66	73.5	93

Table S2. Change of estimated log-likelihood and information criteria (from top to bottom: final refined model, best structural model, and initial starting model).

-2 x log-likelihood (OFV)	2750.86
Akaike Information Criteria (AIC)	2786.86
Bayesian Information Criteria (BIC)	2815.36
Corrected Bayesian Information Criteria (BICc)	2831.57
-2 x log-likelihood (OFV)	2875.04
Akaike Information Criteria (AIC)	2901.04
Bayesian Information Criteria (BIC)	2952.63
Corrected Bayesian Information Criteria (BICc)	2937.84
-2 x log-likelihood (OFV)	3322.92
Akaike Information Criteria (AIC)	3336.92
Bayesian Information Criteria (BIC)	3348.01
Corrected Bayesian Information Criteria (BICc)	3357.27
